# Ocularity Feature Contrast Attracts Attention Exogenously

**DOI:** 10.3390/vision2010012

**Published:** 2018-02-24

**Authors:** Li Zhaoping

**Affiliations:** Department of Computer Science, University College London, London WC1E 6BT, UK; z.li@ucl.ac.uk

**Keywords:** attention, ocularity, visual search, saliency, exogenous guidance, ocularity singletons

## Abstract

An eye-of-origin singleton, e.g., a bar shown to the left eye among many other bars shown to the right eye, can capture attention and gaze exogenously or reflexively, even when it appears identical to other visual input items in the scene and when the eye-of-origin feature is irrelevant to the observer’s task. Defining saliency as the strength of exogenous attraction to attention, we say that this eye-of-origin singleton, or its visual location, is salient. Defining the ocularity of a visual input item as the relative difference between its left-eye input and its right-eye input, this paper shows the general case that an ocularity singleton is also salient. For example, a binocular input item among monocular input items is salient, so is a left-eye-dominant input item (e.g., a bar with a higher input contrast to the left eye than to the right eye) among right-eye-dominant items. Saliency by unique input ocularity is analogous to saliency by unique input colour (e.g., a red item among green ones), as colour is determined by the relative difference(s) between visual inputs to different photoreceptor cones. Just as a smaller colour difference between a colour singleton and background items makes this singleton less salient, so does a smaller ocularity difference between an ocularity singleton and background items. While a salient colour difference is highly visible, a salient ocularity difference is often perceptually invisible in some cases and discouraging gaze shifts towards it in other cases, making its behavioural manifestation not as apparent. Saliency by ocularity contrast provides another support to the idea that the primary visual cortex creates a bottom-up saliency map to guide attention exogenously.

## 1. Introduction

Vision can be roughly decomposed into looking and seeing [1]. Looking is selecting a fraction of visual inputs for deeper processing, e.g., by directing one’s gaze to a particular location so as to put it into one’s attentional spotlight; and seeing is inferring the visual properties from the selected fraction, e.g., to recognize a face within one’s attentional spotlight. Selection is also called attentional selection, and it can be top-down (also called goal-directed or endogenous), such as when directing one’s gaze to the page of a book in order to read, or bottom-up (also called goal-independent, input stimulus driven, or exogenous), such as when gaze is distracted from the page of the book by a sudden movement in the peripheral visual field [2,3]. This paper focuses on the exogenous selection and shows that this selection can be induced by a visual input feature, ocularity, that is often hardly visible to seeing.

In this paper, the strength of a visual location to attract attentional selection exogenously is defined as saliency. For example, a bright spot in a dark field is salient, so is a vertical bar among horizontal bars, a red spot among green ones, or an object moving to the left among many other objects that are static or moving in another direction. Hence, it has been well known that visual locations with a high contrast in visual input features such as luminance or colour, orientation, or motion direction are very salient [4,5,6]. Thus, these visual feature dimensions, colour, orientation, and motion direction, are called basic feature dimensions. In other words, a visual location is salient if, in one of the basic feature dimensions, its visual input feature value has a large spatial contrast from the input feature values at neighbouring locations in the scene. Defining input ocularity as the relative difference between the input to the left eye and the input to the right eye for a visual input item, this paper demonstrates that a contrast in input ocularity also makes a visual location salient. In fact, saliency by ocularity contrast can be no less salient than saliency by orientation contrast, and hence, ocularity also constitutes a basic feature dimension.

**Figure 1:**
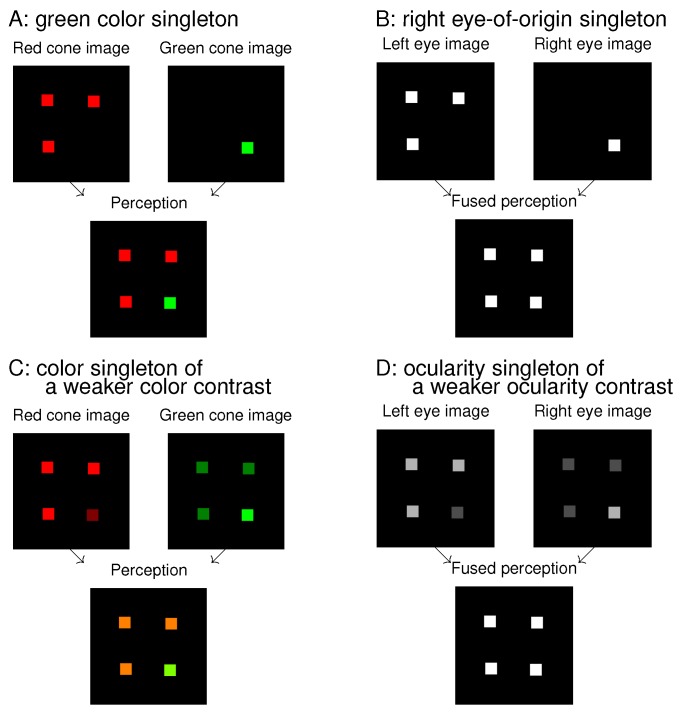
Analogies and differences between colour singletons (**A**,**C**) and ocularity singletons (**B**,**D**). In each of (A–D), two input components are shown at the top and the resulting perception at the bottom; the singleton is the lower right dot in the bottom image. When the inputs to the left eye and right eye are viewed as analogous to inputs to the red cones and green cones, respectively, (B) and (D) are analogous to (A) and (C), respectively. The colour distinctions of the singletons in (A,C) are highly visible perceptually, and the ocularity distinctions of the singletons in (B,D) are not. However, all four singletons can attract attention exogenously.

Reaction time (RT) in a visual search task to find a target is often used to assess saliency [6], such that a shorter RT is associated with a larger saliency at the target’s location. Traditionally, a spatial contrast in input ocularity is not thought to induce a high saliency since ocularity is hardly visible to perception. The invisibility makes it difficult or impossible for one to search for a target defined only by its unique ocularity, and the RT for this search would be very long or indefinite. For example, an input presented to the left eye only and another input presented to the right eye only differ very much in ocularity, but perceptually viewers can barely tell the difference between the two inputs if they are otherwise identical (unless their two eyes differ substantially in, e.g., eye sight). In a previous study, Wolfe and Franzel [7] asked observers to find a monocular white dot (in a dark background) among other white dots presented to the other eye. The target identity would be obvious if observers were allowed to view the visual stimuli by one eye only while closing the other eye. Without this allowance, the input stimuli appeared like that in the image for the fused perception in Figure 1B, and, consequently, the observers were randomly guessing in their responses. A successful performance in such a search task requires that the observer sees or recognizes the target’s distinctive feature, even though the saliency to be assessed by the RT is associated with looking (by exogenous selection) and not with seeing or recognizing.

**Figure 2:**
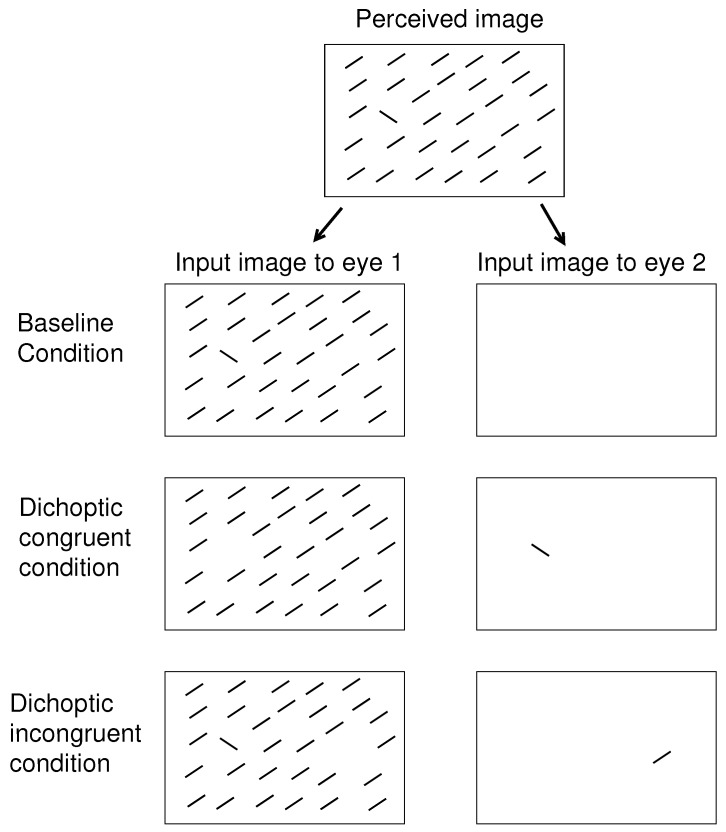
Schematics of the three dichoptic conditions of visual inputs for the visual search task used in the previous [8,9] and the current studies. In this example, the target of the visual search is the uniquely oriented bar. The binocular summation of visual inputs, resembling the perceived image, is the same in these three conditions: baseline, dichoptic congruent (DC), and dichoptic incongruent (DI). In the baseline condition, all the relevant visual items are presented monocularly to the same eye. In the dichoptic congruent (DC) condition, the search target is presented to one eye, and all the non-targets are presented to the other eye; this target is thus an eye-of-origin singleton, or an ocularity singleton. In the dichoptic incongruent (DI) condition, the ocularity singleton is in the right half of the perceived image, while the target is in the left half, or vice versa.

Looking and seeing have different roles, and thus they are likely dissociable. If so, then conceivably, the eye-of-origin singleton like that in Figure 1B could be very salient to attract gaze while its unique eye-of-origin could not be recognized for the search task. To assess its saliency requires a task that does not require observers to recognize the eye-of-origin. For example, one of my previous works [8] involved searching for a uniquely tilted bar in a background of uniformly oriented bars. As illustrated schematically in Figure 2, I presented all bars monocularly and found that the RT to find the target bar was shorter when the target had the unique eye-of-origin (in the dichoptic congruent (DC) condition) compared to when the target shares the same eye-of-origin with all the background bars (in the baseline condition). While observers did not need to recognize the eye-of-origin of the target to perform the task, the target was more salient when its eye-of-origin was unique. I also found that if instead a non-target background bar far from the target had the unique eye-of-origin (in the dichoptic incongruent (DI) condition in Figure 2), the RT became longer compared to when all bars had the same eye-of-origin [8]. This suggested that the non-target singleton in eye-of-origin attracted attention away from the target to interfere with the task. A subsequent experiment with eye tracking confirmed this suggestion: in most trials of the DI condition, the gaze was distracted to the non-target singleton before directing to the target [9].

Figure 1 shows an analogue between colour features and ocularity features. A colour singleton in Figure 1A is analogous to an eye-of-origin singleton in Figure 1B, and they are both salient. This is so even though the colour singleton is distinctive to perception while the eye-of-origin singleton is not. Extending the analogue to that between Figure 1C and Figure 1D, the ocularity singleton in Figure 1D has a weaker ocularity contrast (from the other input items) than the eye-of-origin singleton in Figure 1B, just as the colour singleton in Figure 1C has a weaker colour contrast from the background items than the colour singleton in Figure 1A. As long as the colour contrast is sufficient, the colour singleton is salient, even though its saliency decreases with decreasing colour contrast. Hence, we expect that the ocularity singleton in Figure 1D can attract attention as long as the ocularity contrast is sufficient, even though it may be less salient than the eye-of-origin singleton in Figure 1B. This paper will show that this is indeed the case.

In addition, in a scene of mainly ocularly balanced input items, when one visual item has a large ocular imbalance in its input, i.e., when its inputs to the two eyes differ substantially in input strength, this visual item appears glossy or lustrous and can cause viewers to avoid looking at it. This paper will show that such a perceptual quality of lustre, at the level of seeing rather than looking, could also obscure the behavioural manifestation of the saliency by ocularity contrast. While the invisibility of the eye-of-origin feature obscured the saliency effects of an eye-of-origin singleton in Wolfe and Franzel’s study, visual aversion rather than invisibility obscures the saliency effects by an ocularity singleton due to ocular imbalance.

To reveal saliency effects by ocularity singletons, visual search tasks similar to those in the previous studies [8,9] were used. The task was either to search for a bar with a unique orientation from a background of uniformly oriented bars, or to search for a letter ‘T’ among letter ‘L’s. In either task, the observer had to press as quickly as possible a left or right button on a keypad held by both hands, using the left or the right thumb, respectively, to indicate whether the target was in the left or right half of the search array in their perceived visual image or scene. Let $C_{L}$ and $C_{R}$ be the input contrasts to the left and right eyes, respectively, for each search item, e.g., a bar or a letter. The value of $C_{L}$ or $C_{R}$ is defined as the difference $L-L_{o}$ between the luminance *L* of an input item and the luminance $L_{o}$ of the background (which is uniformly white, black or grey), divided by the maximum possible luminance difference $L-L_{o}$ by the visual display. Thus, $0\leq C_{L}\leq 1$ and $0\leq C_{R}\leq 1$ always. Denoting $s_{L}\equiv C_{L}/\left(C_{L}+C_{R}\right)$ and $s_{R}\equiv C_{R}/\left(C_{L}+C_{R}\right)$ so that $s_{L}+s_{R}=1$ always, the ocularity of an item is defined as $O=s_{L}-s_{R}$. A monocular item has O=1 or O=−1; an ocularly balanced item has O=0; and a left-eye dominant item has a positive *O* while a right-eye dominant item has a negative *O*. The dichoptic condition of the visual inputs could be baseline when all the search items have the same ocularity *O*. It can be DC, when all the search items have the same ocularity except for the target, or can be DI, when all search items have the same ocularity except for one non-target item in the opposite lateral half of the search array (in the perceived scene) from that of the target; see Figure 2 for an example.

In all the experiments in this study, the search target was never defined by its ocularity value, so the dichoptic condition was irrelevant to the visual search task. If the ocularity singleton is salient to attract attention exogenously, then the baseline, DC, and DI conditions give three different attentional cueing situations for the task: uncued, validly cued, and invalidly cued, respectively. In other words, while the baseline condition has no ocularity singleton to cue attention, the ocularity singleton in the DC and DI conditions, respectively, cues attention towards and away from the target. Some experimental sessions randomly interleaved trials from all the three dichoptic conditions; others randomly interleaved just the baseline and the DC conditions. The RTs for the three dichoptic conditions will be denoted as $RT_{\mathrm{baseline}}$, $RT_{\mathrm{DC}}$ and $RT_{\mathrm{DI}}$, respectively. A relative ease of the task in the DC compared to the DI conditions, e.g., a positive $RT_{\mathrm{DI}}-RT_{\mathrm{DC}}$, is a cueing effect or a behavioural manifestation of the saliency by the ocularity singleton, in one of the usual manners to assess attentional attraction by contrasting the validly cued and invalidly cued situations. Sometimes, but not always, the saliency of the ocularity singleton can also be manifested as a positive $RT_{\mathrm{baseline}}-RT_{\mathrm{DC}}$, the ease of the task in the DC condition relative to that in the baseline condition (cf. validly cued versus uncued), or as a positive $RT_{\mathrm{DI}}-RT_{\mathrm{baseline}}$ (cf. invalidly cued versus uncued). The saliency can also be manifested as the priority to saccade towards the ocularity singleton before saccading elsewhere, when gaze is tracked during the search. Some results in this paper have been presented previously in preliminary forms [10,11].

## 2. Materials and Methods

All experiments, Experiments 1–4, in this study were adapted from previous experiments in terms of equipment, stimulus designs, and visual tasks. Hence, I will briefly outline the essentials and highlight the differences, since more details can be found in published papers [8,9]. In this paper, each reported luminance of visual inputs always refers to the luminance measured directly from the visual display, without taking into account the luminance reduction along the optical pathway from the display to the eyes by the presence of the stereo goggles, the mirror stereoscope, and/or the half-reflective mirror for the eye tracker. For example, in Experiment 1, the actual luminance reaching the eyes was reduced by seven eighths by the pair of stereo goggles used to view the dichoptic stimuli.

### 2.1. Experiment 1

**Figure 3:**
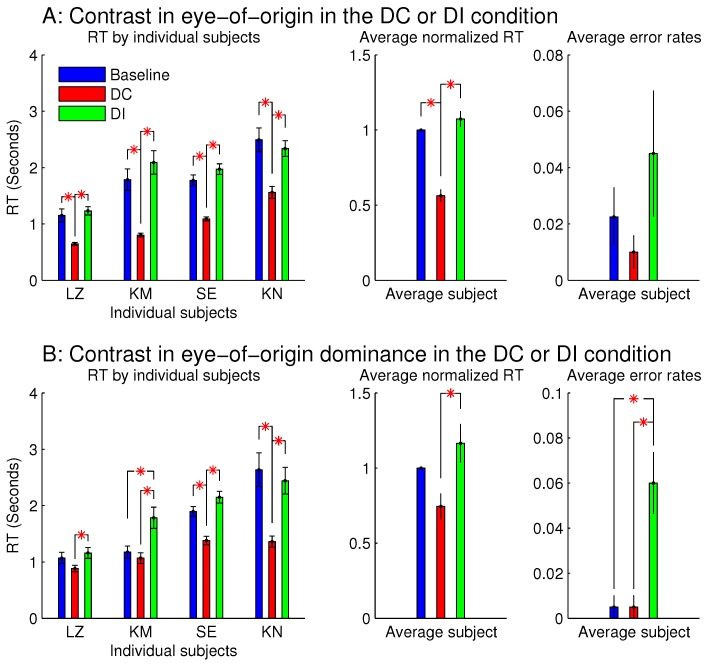
Reaction times (in button press responses) and error rates (the number of incorrectly responded trials as a fraction of the number of all the trials in a given condition) in the task to search for a uniquely oriented bar in a background of uniformly oriented bars (all bars were tilted 10 deg, clockwise or anti-clockwise from horizontal). Stimuli were displayed on a CRT, viewed by a pair of stereo goggles. The attentional capture by the eye-of-origin singletons (**A**) is extended to the capture by the singletons in eye-of-origin dominance (**B**). In the latter, for all the bars, the input contrast to the input-dominant eye was nine times that to the other eye (i.e., $s_{L}=9s_{R}$ or $s_{L}=s_{R}/9$). For (B), each search item had a random disparity within a range of $\left(-0.3^{\circ },0.3^{\circ }\right)$, except for the zero-disparity items in the left-most and right-most columns of the search array. In each figure of this paper, all error bars mark the standard errors of the means, and a red ‘*’ linking two data bars indicates a significant difference (p<0.05) between the two linked data points.

Experiment 1 extends from Experiment 4 of [8], and the main text highlights the main difference between the two studies. All stimulus bars were $0.12^{\circ }\times 1.1^{\circ }$ bright rectangles in a black background, displayed on a Clinton Monoray cathode ray tube (CRT) at a frame rate of 150 Hz, viewed at a distance of 40 cm in a dim room using a pair of FE-1 shutter goggles from the Cambridge Research System. The target bar was tilted $10^{\circ }$ from horizontal, in the clockwise or counter-clockwise direction with equal probability, and the non-target bars were tilted $10^{\circ }$ from horizontal in the other direction. The bars formed an array of 22 rows by 30 columns, extending $34^{\circ }\times 46^{\circ }$ in visual space, and each bar’s location was randomly and independently displaced from its regular grid position by up to $0.24^{\circ }$ horizontally and vertically. The target’s location was about $15^{\circ }$ (at least $12^{\circ }$ horizontally) from the centre of the array. A task-irrelevant bright binocular dot ($0.12^{\circ }\times 0.12^{\circ }$) was placed at the centre of mass of every four closest neighbouring bars, and a task-irrelevant binocular disk ($0.5^{\circ }$ diameter) was also placed at each of the four corners outside the array of bars. These binocular dots and disks were used to anchor binocular vergence. The dots were displayed simultaneously with the stimulus bars while the disks were constantly on the display throughout each experimental session. The dichoptic inputs $C_{L}$ and $C_{R}$ for each bar was such that $C_{L}+C_{R}$ were fixed for all bars, while $s_{L}=C_{L}/\left(C_{L}+C_{R}\right)$ varied with experimental conditions and trials. When $s_{L}=s_{R}=0.5$, the bar was 24 ${\mathrm{cd}/m}^{2}$ on the screen without wearing the stereo shutter goggles. In the session of Figure 3B, each bar (except for the zero-disparity items in the left-most and right-most columns) had a random horizontal disparity independently chosen from the range $\left(-0.3^{\circ },0.3^{\circ }\right)$ by shifting the bar’s locations in the two monocular images by an equal amount (half of the disparity magnitude) in the opposite horizontal directions. The central fixation point ($0.3^{\circ }$ diameter disk) was binocular and presented for about 1.2 s on a blank screen (other than the four corner disks to help anchor vergence) before the bar stimulus onset. This fixation screen was triggered by the subject’s button press to start a trial, and the bar stimulus disappeared after the subject’s button response for the target’s location. In each session, there were 50 trials for each condition for each subject, except for subject LZ, who had only 20 trials for each condition.

### 2.2. Experiments 2–4

These three experiments were adapted from Experiment 1b in a previous eye tracking study [9], using a set up with a mirror stereoscope (in front of a Mitsubishi 21-inch CRT display at a frame rate of 100 Hz) and a video eye tracker schematically shown in Figure 4A of [9]. All the eye-tracking techniques and details (such as the criteria for fixations and saccades) are as in [9] unless mentioned otherwise. The right eye of the observers was tracked.

In Experiment 2, the bars were in a 23×23 array extending $18.4^{\circ }\times 18.4^{\circ }$. Each bar was $0.6^{\circ }\times 0.085^{\circ }$, tilted $45^{\circ }$ from horizontal. The locations of the bars were randomly and independently jittered from the respective regular grid positions by up to $0.05^{\circ }$ horizontally and vertically. The possible grid locations for the target in the search array were within Rows 4–20, closest on a circle that was centred on the array and had a radius of 10 grid units. Each target was at least 6 grid units horizontally from the centre of the array. Each observer participated in one session, which had 450 trials, with 50 trials for each condition.

In Experiment 3, the letters were each $0.7^{\circ }\times 0.7^{\circ }$ in size, and each horizontal or vertical stroke of the letters had a thickness of $0.085^{\circ }$. The locations of the letters were randomly jittered from the regular grid positions by up to $0.064^{\circ }$ horizontally and vertically. The search array was 17×17, extending $18.6^{\circ }\times 18.6^{\circ }$. The possible grid locations for the target in the search array were within Rows 4–14, closest on a circle that was centred on the array and had a radius of 7 grid units. Each target was at least 5 grid units horizontally from the centre of the array. Each observer participated in one session, which had 400 trials, with 50 trials for each condition.

In Experiment 4, the letters were in a 14×14 array (extending $18.3^{\circ }\times 18.3^{\circ }$), each letter was $0.75^{\circ }\times 0.75^{\circ }$ in a sans-serif font. The locations of the letters were randomly jittered from the regular grid positions by up to $0.077^{\circ }$ horizontally and vertically. The target was in Rows 4–11, at a horizontal distance of 2–5 grid units from the centre of the array, and within a radius of 3–6 grid units from the centre of the array. Each observer participated in one session, which had 45 search trials for each condition. The eye tracking data in this experiment had poor quality or were incomplete for several observers and hence were not analysed further.

In Experiment 2 and Experiment 3, each bar or letter in the search array was dark in a white background of $110\;{\mathrm{cd}/m}^{2}$, and its luminance was $110\cdot \left(1-s_{L}\right)\;{\mathrm{cd}/m}^{2}$ for the left eye and $110\cdot \left(1-s_{R}\right)\;{\mathrm{cd}/m}^{2}$ for the right eye with $0\leq s_{L}\leq 1$, $0\leq s_{R}\leq 1$, and $s_{L}+s_{R}=1$ defining the relative strength of left eye and right eye inputs. The values of $s_{L}$ and $s_{R}$ were assigned according to experimental conditions as described in the main text.

In Experiment 4, each letter in the search array was lighter than the grey background, unlike Experiments 2 and 3. In addition, each letter also had an additional random luminance fluctuation. For each zero-ocularity non-target letter, the monocular luminance of the strokes in the letter was $L_{o}+L_{1}\cdot \left(0.5+x\right)$ where $L_{o}=43\;{\mathrm{cd}/m}^{2}$ was the background luminance, and $L_{1}=67\;{\mathrm{cd}/m}^{2}$, and *x* was a random number within (−0.3,0.3) for each letter (identical for the two monocular images) and was independent between letters. For the target letter, the monocular luminance of the strokes in the letter was $L_{o}+L_{1}\cdot s\cdot \left(1+x\right)$, where $s=s_{L}$ or $s_{R}$ for the left or right monocular image, and *x* (identical for the two monocular images) was a random number within the range (−0.2,0.2).

In each of Experiments 2–4, the disparity of each search item (except for the zero-disparity items in the left-most and right-most columns) was randomly and independently assigned to within the range of $\left(-0.21^{\circ },0.21^{\circ }\right)$ by shifting the horizontal positions of each monocular image item in a similar way as that in the session depicted by Figure 3B for Experiment 1. In addition to the binocular dots (which were square dots with side length $0.17^{\circ }$) between the search items to anchor vergence, the monocular images of the search arrays were enclosed by a vergence anchoring frame, which was black for Experiments 2–3 and white for Experiment 4, like that illustrated in Figure 4B of [9]. The size of this anchoring frame was 10% larger than the extent of the search array.

### 2.3. Data Analysis

For each observer and each stimulus condition, the RT of the mean button press was the average of the RTs for the button presses across the pool of the trials of this observer and this condition. This pool excludes the trials in which the button press was incorrect and excludes the trials in which the button press RTs were outliers. A button press RT was an outlier if it was shorter than 0.2 s or longer than the average RT by three standard deviations of the RTs (with the average and standard deviation calculated before removing the RT outliers) for this observer and this condition. This same pool of trials was also used to calculate the average RT for the gaze to reach the target (excluding in addition trials in which the gaze did not reach the target).

The average RT or error rate across subjects were the average of the mean RTs or error rates, respectively, of individual subjects. All error bars in the plots were standard errors of the mean, whether it was the mean across the trials for a subject or the mean across observers. A significant difference between two quantities was when the *p*-value in a *t*-test satisfies p<0.05. Matched (subject) sample *t*-tests were used when comparing two conditions involving the same set of subjects. When RTs vary too much between observers, the RTs were normalized by dividing each subject’s RT for a given condition by this subject’s RT for the corresponding baseline condition, before averaging across subjects. These results are then plotted out as the normalized RTs.

## 3. Results

The first experiment, Experiment 1, extends directly from Experiment 4 in the original study [8] that demonstrated the saliency of an eye-of-origin singleton. The task for the observers was to search for a uniquely oriented bar and report as soon as possible by button press whether the target was in the left or right half of the array of bars in their perceived image. All stimulus bars were bright on a black background, tilted $10^{\circ }$ or $-10^{\circ }$ from horizontal. The target bar and non-target bars were tilted in opposite directions such that there was a $20^{\circ }$ orientation contrast between the target and non-target bars. This experiment copied the following aspects from Experiment 4 in the original study [8]: equipment setup, the observer’s task, and the properties of the visual stimuli, in terms of the size (22 rows and 30 columns extending over a $34^{\circ }\times 46^{\circ }$ visual space) of the search arrays, the size of the stimulus bars (each a $0.12^{\circ }\times 1.1^{\circ }$ rectangle), the task-irrelevant binocular dots to anchor vergence, the central fixation point before the search array onset, and the viewing distance (40 cm). It differed from the previous design in the following aspects: (1) a larger (doubled) extent of the random position jitters of the search items, (2) the angle ($\pm 10^{\circ }$, rather than $\pm 25^{\circ }$) of each bar from horizontal, and (3) the dichoptic input properties of the search items. Aspects (1) and (2) made the target bar less salient by the spatial, non-dichoptic, properties of the visual stimuli and should prolong the RT to perform the task. The third aspect was to alter input dichoptic properties session by session to investigate how saliency depended on these properties. In each session, each trial was equally likely to be a baseline, DC, or DI trial. Each of the four observers had normal depth vision and participated in all the sessions of this experiment in a random order. One observer (LZ) was the author, while the other participants were naive to the purpose of the study (although one (KM) of them could perhaps guess the purpose since he was familiar with the research interests of the author).

### 3.1. From Eye-Of-Origin Singletons to Ocular Dominance Singletons

In one session of Experiment 1, all bars were monocular like in the previous study [8], so that the ocularity singleton was an eye-of-origin singleton. In each trial, the eye of origin of the target bar was randomly the left or the right eye. In another session, each bar was shown to both eyes, but its luminance (43.2 ${\mathrm{cd}/m}^{2}$) in one eye was nine times as strong as its luminance (4.8 ${\mathrm{cd}/m}^{2}$) in the other eye, making the bar left-eye dominant or right-eye dominant in its input. In the baseline dichoptic condition, all the bars for a given trial had the same dominant eye. In the DC or DI condition, the ocularity singleton had one dominant eye and the other bars had the other dominant eye. In each trial, the target bar’s dominant eye was randomly the left or the right eye with equal chance.

Figure 3 shows the observations from these two sessions. When all the stimulus bars were monocular so that the ocularity singletons were eye-of-origin singletons (Figure 3A), $RT_{\mathrm{DC}}$ was significantly shorter than $RT_{\mathrm{baseline}}$ and $RT_{\mathrm{DI}}$. This indicates that the target was more salient when it was also the eye-of-origin singleton; this is like the findings from the previous study [8]. However, $RT_{\mathrm{DI}}$ was not significantly longer than $RT_{\mathrm{baseline}}$, unlike the finding from the previous study [8], perhaps because the RTs in this study were about twice as long as those in the previous study mainly due to the smaller orientation contrast between the target bar and the background bars. Conceivably, if the ocular singleton distracted attention in the DI condition away from the target so that it consumed approximately an extra 200 millisecond (ms) for the task completion, this extra duration would be more noticeable out of an $RT_{\mathrm{baseline}}\approx 500$ ms, rather than an $RT_{\mathrm{baseline}}\approx 1000$–2000 ms. In any case, the significant difference between $RT_{\mathrm{DC}}$ and $RT_{\mathrm{DI}}$ is a signature of the saliency by the eye-of-origin singleton. The error rate for the DI condition was slightly although not significantly larger than those in the DC and baseline conditions. It is likely that the observers in their hurried decisions were more likely to make an error by mistaking the salient non-target ocular singleton as the target.

When each stimulus bar was left-eye or right-eye dominant, rather than monocular, qualitatively similar results were observed (Figure 3B). Hence, saliency by an eye-of-origin singleton can be extended to saliency by an ocularity singleton, at least in this particular stimulus situation when the ocularity singleton was an eye-dominance singleton and when all the search items had the same magnitude $\left|O|=\right|s_{L}-s_{R}|$ of ocularity.

### 3.2. An Ocularity Singleton Is More Salient When It Has a Larger Ocularity Contrast from Background Inputs

Another experiment, Experiment 2, further investigated the saliency by ocularity singletons by varying the ocularity contrast between the singleton and the background items. This experiment is very similar to an experiment (Experiment 1b) in a previous eye tracking study [9] in the equipment setup (using a mirror stereoscope and a video eye tracker), the task and the visual stimuli (including the binocular dots between the search items to anchor vergence). Like in Experiment 1, the observer’s task was to search for a uniquely oriented bar among uniformly oriented background bars, and each trial was randomly a baseline, DC, or DI trial with equal chance. Each bar was a $0.6^{\circ }\times 0.085^{\circ }$ rectangle, tilted $\pm 45^{\circ }$ from horizontal, and the target bar was tilted in a unique direction from horizontal. The bars were dark in a white background (of 110 ${\mathrm{cd}/m}^{2}$), and they formed a 23×23 array (with some random spatial jitters from the regular grid) extending to a visual space of $18.4^{\circ }\times 18.4^{\circ }$. Each bar in a search array had a random disparity value within a range of $\left(-0.21^{\circ },0.21^{\circ }\right)$, except for the zero-disparity bars in the right-most and left-most columns of the search array. Six observers participated; all had normal depth vision, and all except one were naive to the purpose of the study.

The input contrasts $C_{L}$ and $C_{R}$ for the left and right eyes for the stimulus bars were such that $C_{L}+C_{R}$ was held constant across all the bars within a trial and across the trials within a session. Meanwhile, the ocularity magnitude $\left|O|=\right|s_{L}-s_{R}\left|=\right|C_{L}-C_{R}|/\left(C_{L}+C_{R}\right)$ was constant across the bars within a trial, but varied randomly across trials to be |O|=1/5, 1/3, or 3/5. In a DC or DI trial, the ocularity singleton had the unique sign of the ocularity *O* (i.e., the unique dominant eye) among all the bars. Hence, each session randomly interleaved trials from nine different conditions arising from all the combinations of the three ocularity magnitudes |O| and the three possible dichoptic conditions (baseline, DC, and DI).

**Figure 4:**
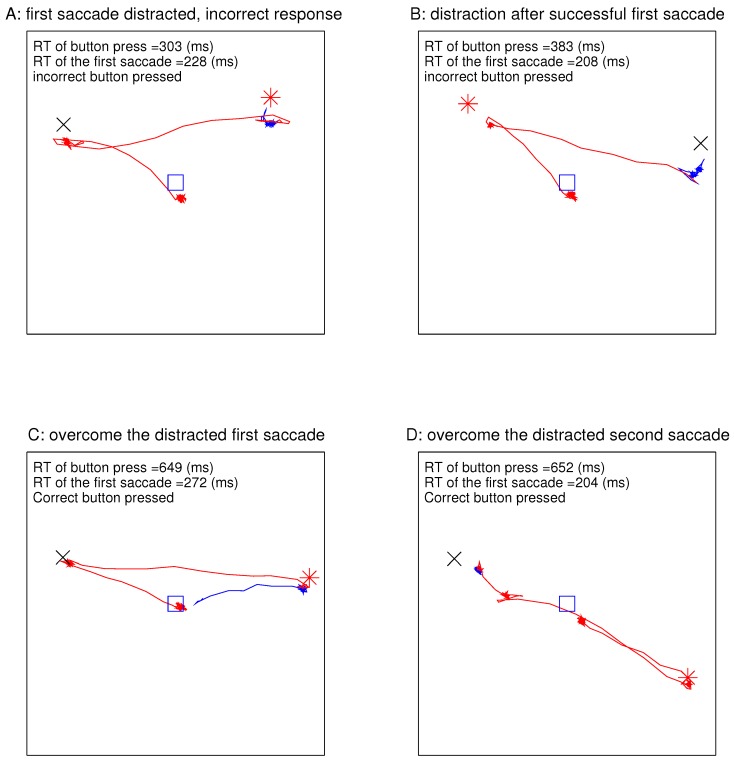
Gaze traces in four example DI trials (of Experiment 2), when an observer searched for an orientation singleton bar in the presence of a distracting ocularity singleton in the search array. Each box frames the region of the perceived visual field containing the array of bars. The locations of the central fixation point (before the stimulus onset), the target, and the distracting ocularity singleton are marked by the blue □, the red *, and the black ×, respectively. The red and blue traces are gaze trajectories before and after the button press, respectively. In each frame, the RT of the first saccade during the search, the RT of the button press, and whether the correct or incorrect button was pressed are indicated.

Figure 4 shows the eye traces in four example DI trials with the largest magnitude |O|=3/5 for the largest ocularity contrast between the ocularity singleton and the other bars. In each example, the gaze started at the central fixation point at the start of the stimulus onset, since the eye tracker verified that the gaze was directed centrally before the search array onset for each trial. In Figure 4A, the first gaze shift occurred at 228 ms after the stimulus onset and was distracted to the non-target ocularity singleton, and the incorrect button was pressed at 303 ms (a very short RT) from the stimulus onset even though gaze went toward the target by the time the button was pressed. Due to the latency for executing a motor command, it was likely that, in this trial, the decision to press the button was made before the saccade towards the target. In Figure 4B, the first saccade was in the correct direction towards the target, but the incorrect button was pressed at an RT of 383 ms upon the gaze reaching the distractor. In Figure 4C, the gaze distraction in the first saccade was overcome so that the correct button was pressed at an RT of 648 ms after the subsequent saccade brought the gaze to the target. In Figure 4D, the gaze went first towards the target before turning towards the distractor, and the correct button was pressed after the gaze had reached the distractor.

**Figure 5:**
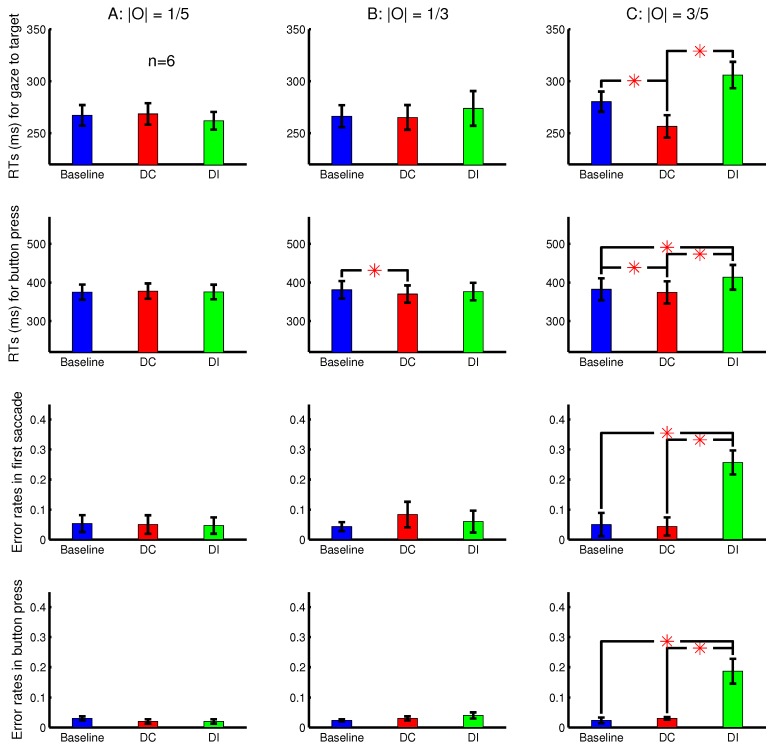
The attentional attraction by the ocularity singleton bar increases with the ocularity contrast between this singleton bar and other input bars. Shown here are behavioural results averaged over n=6 observers in the task to search for a uniquely oriented bar in 23 rows by 23 columns of bars, with the target bar and non-target bars tilted $45^{\circ }$ in opposite directions from vertical. All the bars in a single trial shared the same ocularity magnitude |O|, which can be weak (**A**) (left column), intermediate (**B**) (middle column), or strong (**C**) (right column). The ocularity singleton (in a DC or DI trial) had the unique sign of this ocularity $O=s_{L}-s_{R}$ among the bars in a trial, making the ocular contrast 2|O|. Shown are the RTs for the gaze to reach the target (top row) or for the button press (second row) and the error rates in the first saccade (third row) and button press (bottom row). Each subject’s data were from an experimental session in which the trials from the nine conditions (baseline, DC, and DI in each of (A–C)) were randomly interleaved. The first saccade during a search was deemed erroneous when it went to the left or right from the central fixation (in the perceived image) when the target was to the right or left, respectively.

Figure 5 shows the RTs, averaged across six observers, for the gaze to reach the target and for the correct button presses in the nine conditions. It also shows the error rates of the button presses and the error rates in the lateral direction of the first saccade. The first saccade is defined as erroneous when the target was in the right or left half of the search array in the perceived image while this saccade went (from the central fixation point) leftward or rightward, respectively. The shorter RTs and fewer errors in the DC than the DI trials are again signatures of the saliency of the ocularity singleton. With eye tracking, another signature is the direction of the first saccade reporting the priorities of visual locations for visual selection. In this experiment, these signatures are apparent only when the ocularity contrast 2|O|=6/5 between the ocularity singleton and the other input bars was the largest.

The button presses had an $RT_{\mathrm{baseline}}\approx 400$ ms, which is shorter in this Experiment 2 than that $RT_{\mathrm{baseline}}\approx 1000$ ms in Experiment 1. This is mainly because Experiment 2 had (1) a larger orientation contrast between the target bar and non-target bars and (2) a smaller spatial extent for the search array. The shorter $RT_{\mathrm{baseline}}$ made the difference $RT_{\mathrm{DI}}-RT_{\mathrm{baseline}}$ more apparent when $RT_{\mathrm{DI}}-RT_{\mathrm{DC}}$ was significant. In comparison, in Experiment 1, $RT_{\mathrm{DI}}-RT_{\mathrm{baseline}}$ was not significant and negligible. In other words, the distracting effect of the ocularity singleton, measured by $RT_{\mathrm{DI}}-RT_{\mathrm{baseline}}$, was more noticeable in Experiment 2 in which the overall RTs were shorter. This distracting effect was also apparent in the previous study [8] in which the button press $RT_{\mathrm{baseline}}\approx 600$ ms. Meanwhile, since RTs cannot be shorter than a certain minimum floor value, the cueing effect measured by a significantly positive difference $RT_{\mathrm{baseline}}-RT_{\mathrm{DC}}$ is not as substantial here in Experiment 2 as it was in Experiment 1.

When the ocularity singleton was sufficiently salient, the error rate for the first saccade direction during search was larger in the DI than the other conditions. This error rate was much less than 50% in this study, like that in Experiment 1b of [9]. However, this error rate could be more than 50% when the search array was more spatially extended and when the salient singletons are more eccentric, as in Experiment 1a of [9].

**Figure 6:**
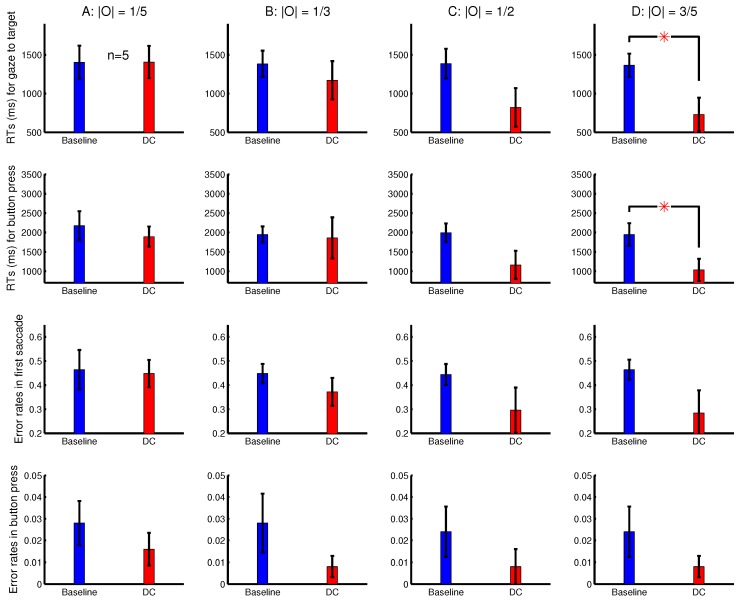
Another experiment (Experiment 3) showing increased saliency of the ocularity singleton by its increased ocularity contrast. Shown are the average behaviour from n=5 subjects in the task to search for a letter ‘T’ among letter ‘L’s in 17 rows by 17 columns of letters. The ocularity magnitude |O| was weak (**A**), medium-low (**B**), medium-high (**C**), or strong (**D**) in each trial and was the same across all the letters in a single trial. The ocularity $O=s_{L}-s_{R}$ of the ocularity singleton (in a DC trial) was the negative of the ocularity for all the other letters in the trial. Each subject’s data were from an experimental session in which the trials from the eight conditions (baseline and DC for each of (A–D)) were randomly interleaved.

To observe a more substantial RT difference $RT_{\mathrm{baseline}}-RT_{\mathrm{DC}}$ when the ocularity singleton is sufficiently salient, another eye tracking experiment, Experiment 3, used a task in which $RT_{\mathrm{baseline}}$ was longer. It was like Experiment 2, but the task was to search for a target letter ‘T’ among non-target letter ‘L’s. This task is harder since the target does not have a uniquely oriented bar to make it salient in the baseline condition. To focus on $RT_{\mathrm{baseline}}-RT_{\mathrm{DC}}$, Experiment 3 omitted the DI trials and added additional conditions with |O|=1/2. Each experimental session randomly interleaved eight conditions, made from all the combinations of four possible |O| values (1/5, 1/3, 1/2, and 3/5) and two dichoptic conditions (baseline and DC). Figure 6 shows the RTs and error rates in Experiment 3 averaged across five observers. For the conditions with a larger |O|=3/5 and |O|=1/2 (and thus a larger ocularity contrast), the difference $RT_{\mathrm{baseline}}-RT_{\mathrm{DC}}$ was substantial, but was only significant when |O|=3/5 (due to a large variability across observers). This was so both for the RT of gaze arrival to the target and for the RT of correct button presses. In Experiment 3, four out of the five observers were naive to the purpose of the study, and the sixth observer was removed from data analysis because he could not see depth well (qualitatively, the same results would be obtained if this observer’s data were included).

### 3.3. Asymmetry between Ocularly Balanced and Ocularly Unbalanced Items

**Figure 7:**
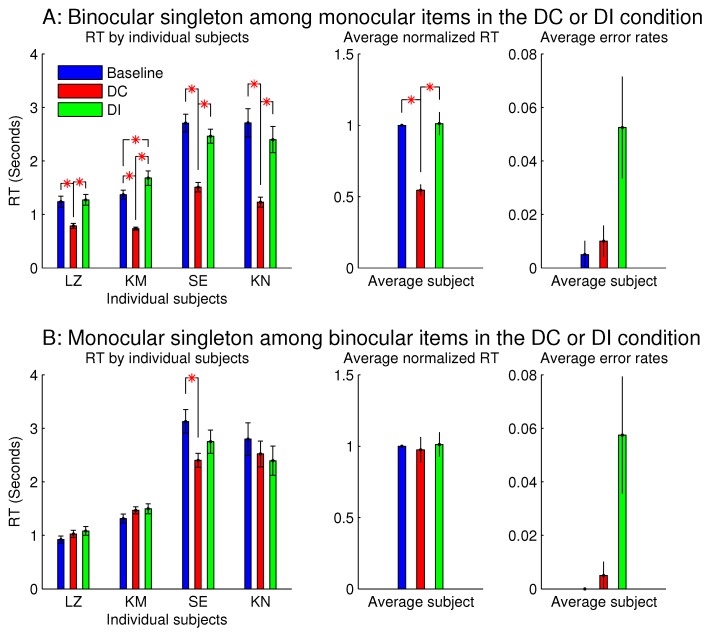
This is like Figure 3, but it contrasts between one ocularity singleton that was a binocular item among monocular items and another that was a monocular item among binocular items. Data for the two figures were collected from separate experimental sessions involving the same task and the same subjects.

Figure 7 shows the observations from another two sessions in Experiment 1, which involved orientation singleton search and randomly interleaved the baseline, DC, and DI trials in each session. In one of these two sessions, all the bars were monocular except for the ocularity singleton, which was binocular, while the binocular summation $C_{L}+C_{R}$ of inputs was constant across all the bars. In this session (see Figure 7A), the saliency of the ocularity singleton was apparent, and both $RT_{\mathrm{baseline}}-R_{DC}$ and $RT_{\mathrm{DI}}-R_{DC}$ were significantly positive. In the other session (Figure 7B), all the bars were binocular, except for the ocularity singleton, which was monocular (again with $C_{L}+C_{R}$ held constant across the bars), and $RT_{\mathrm{DI}}-RT_{\mathrm{DC}}$ was insignificant in any observer or averaged across the observers. However, these two sessions were identical in terms of the ocularity contrast between that of the ocularity singleton bar and that of the other bars. Interestingly, the error rates in button presses were highest, though not statistically significant, in the DI condition in both sessions. This hints that, when the ocularity singleton was a monocular item among binocular items, this singleton may still distract attention when it was a non-target even though it did not reduce $RT_{\mathrm{DC}}$ when it was the target.

The author, who was one (LZ) of the subjects, observed that the monocular singleton was highly conspicuous since it appeared shiny or much brighter than other search items, as if it was an illuminant light source. Furthermore, she noticed that this shiny appearance made her instinctively avert her gaze from it as if to avoid looking at the Sun. Another observer reported that the brightest item in the search array was often not the search target in half of the trials, and that this perhaps inhibited him from looking at this brighter item when it was actually the target. Spatially local imbalance between inputs to the two eyes has been observed to cause the perception of lustre or glossiness [7,12,13,14,15,16]. This appearance is comprehensible since a specular surface could reflect a light source in a very directional manner, so that the reflected light could reach one, but not the other, eye of the viewer, causing ocular imbalance of inputs, which can then be used to infer the shiny or glossy nature of the object surface. Perhaps the monocular singleton distracted attention in the DI trials because it was highly conspicuous and that, when it was also the search target in the DC trials, it did not reduce the $RT_{\mathrm{DC}}$ because the observers felt inhibited from shifting gaze towards it. This is another example when an item’s appearance interfered with the behavioural investigation of its saliency. In this example, the appearance is lustre or glossiness by local ocular input imbalance; in Wolfe and Franzel’s study [7], the appearance is the invisibility of the eye-of-origin feature.

**Figure 8:**
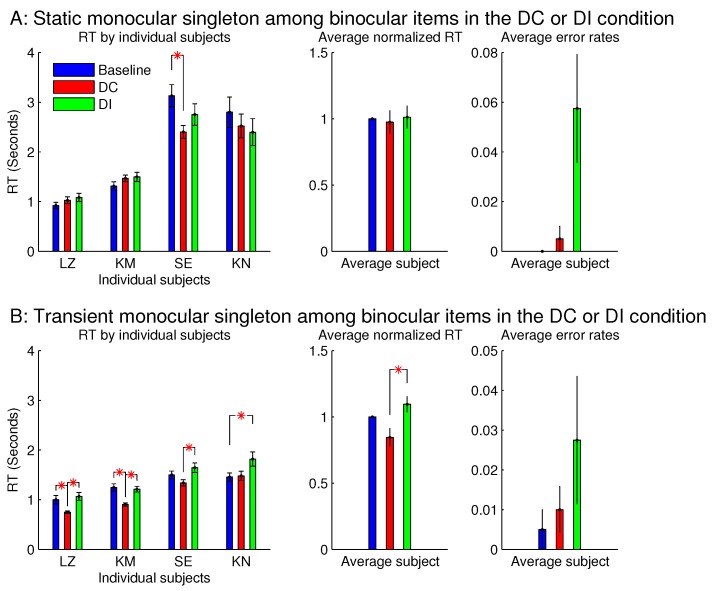
A transient ocularity contrast also attracted attention; and the transientness helped to reveal the attentional attraction by a monocular singleton in a background of binocular items. (**A**) are plots from Figure 7B and (**B**) are plots from another session that differed from the session in (A) by making the ocularity contrast transient. Specifically, the monocularity of the singleton was present only in the initial 0.15 s of the search stimulus, and after this initial period, this search item became binocular (while $C_{L}+C_{R}$ was unchanged) like all the binocular items in the search display.

Figure 8 showed that the saliency of the monocular singleton can be unmasked in another session in which the ocularity contrast was transient so that it was present only in the initial 0.15 seconds (s) of the search stimulus display. In other words, after the initial 0.15 s, the monocular singleton became binocular while keeping the binocular input summation $C_{L}+C_{R}$ unchanged and identical to that of the other bars in the search array. Now, averaged across the same set of four observers, $RT_{\mathrm{DC}}$ was significantly shorter than $RT_{\mathrm{DI}}$. Although the ocularity contrast was brief, it was sufficient to attract attention since exogenous attraction to attention acts quickly [17,18].

However, since seeing the properties (e.g., lustre) of a visual object usually occurs after looking (overtly or covertly via attentional guidance), the briefness of the monocularity of the input item was likely to reduce or even prevent the perception of lustre, thereby reducing or preventing the interference by this percept.

However, for sessions with the binocular singleton bar among monocular bars, making the ocularity contrast transient did not qualitatively change the RTs or the error rates.

Baker and colleagues [19] observed that the perceived luminance contrast of an input item is often a nonlinear summation of the input luminance contrasts $C_{L}$ and $C_{R}$ to the two eyes, so that given a fixed $C_{L}+C_{R}$, the perceived contrast is often larger when the inputs are ocularly unbalanced $C_{L}\neq C_{R}$. The strongest nonlinearity is when the perceived luminance contrast is max($C_{L},C_{R}$), the larger one of $C_{L}$ and $C_{R}$, and this is approximately the case when visual inputs are luminance decrements from the background luminance. In contrast, a lack of nonlinearity is when $C_{L}+C_{R}$ is the perceived luminance contrast, and this is approximately the case when visual inputs are luminance increments from the background luminance, unless $C_{L}/C_{R}$ is very different from unity. Experiment 1 used luminance increments as visual inputs, but its monocular bars had $C_{L}/C_{R}=0$ or $C_{L}/C_{R}=\infty$. Hence, given the same $C_{L}+C_{R}$ for our monocular and binocular bars, the monocular bar should appear stronger in luminance contrast. It is thus unclear whether the lustrous percept arose from the stronger perceived luminance contrast or from the ocular imbalance of the visual inputs in the context of ocularly balanced inputs.

**Figure 9:**
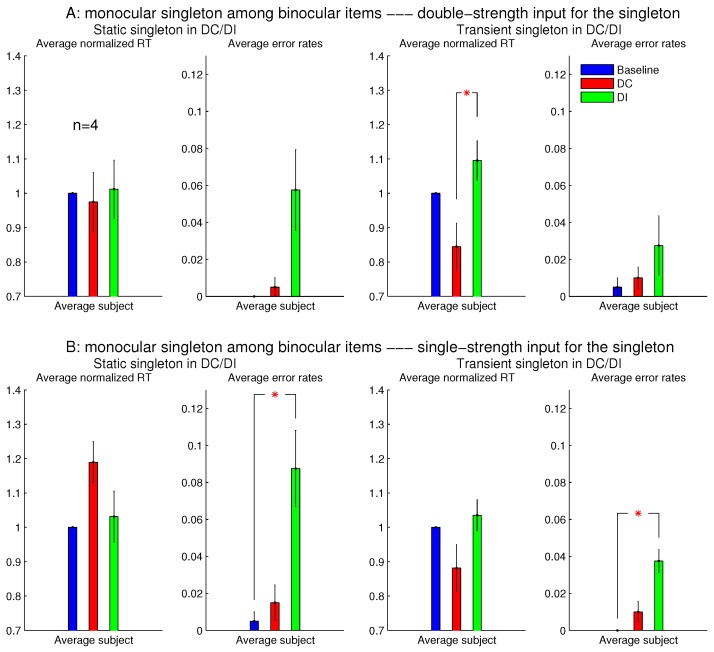
The inhibiting effect of the monocular singleton among binocular items was present even when the monocular item (for which $C_{L}=0$ or $C_{R}=0$) had its monocular input strength max$\left(C_{L},C_{R}\right)$ identical to max$\left(C_{L},C_{R}\right)$ for the binocular items (for which $C_{L}=C_{R}$). The top (**A**) repeats the results (averaged across observers) of the two sessions in Figure 8 for monocular singletons among binocular items, when max$\left(C_{L},C_{R}\right)$ for the monocular singleton was twice that for the binocular items; the bottom (**B**) plots the corresponding results for another two sessions, which are the same as in (A), except that max$\left(C_{L},C_{R}\right)$ for the monocular singleton was equal to max$\left(C_{L},C_{R}\right)$ for the binocular items. The left two columns are for sessions when the ocularity contrast for the singleton was static, and the right two columns are for sessions when this ocularity contrast was transient (for 0.15 s). All four sessions were from Experiment 1 involving the same n=4 observers.

Experiment 1 had two additional sessions involving monocular singletons among binocular bars. They were identical to the two sessions in Figure 8, except that the monocular singleton bar had its max($C_{L},C_{R}$) identical to the max($C_{L},C_{R}$) for the binocular bars, which had $C_{L}=C_{R}$, i.e., for the monocular bar, the luminance contrast presented to one eye only was identical to the luminance contrast for each eye in all the binocular bars. Figure 9 shows that these single-strength monocular singletons (in Figure 9B) were still distracting in the DI trials and were not helpful to reduce the RTs in the DC trials. The author as a subject observed that their evoked lustrous percept was roughly as conspicuous and uncomfortable as that evoked by the double-strength monocular singletons shown in Figure 8 and Figure 9A.

### 3.4. Effects of Depth, Luminance Contrast, and Duration of the Ocularity Contrast between an Ocularly Unbalanced Target among Ocularly Balanced Non-Targets

We have seen that the reaction time to find the target can be affected by the ocularity contrast between the target and the background items and by an observer’s aversion to a target’s perceived lustrous nature. Figure 8 and Figure 9 suggest that the aversion to the lustrous property can be reduced by making the ocularity contrast brief, so as to prevent the slower process for perception (for lustre) while allowing the faster process for saliency. However, the ocularity contrast was made brief by removing it abruptly during the search. Presumably because of this abrupt input change, three out of the four observers reporting seeing blinking or movement associated with an input item in the search array in some of the trials. Since this perceived input change could also serve as a cue to attract attention, it is unclear whether the observed attentional cueing effect was due to the saliency by the ocularity contrast or to the perceived sudden change.

Additionally, when a visual input item is viewed by both eyes in natural vision, the depth of this item can also affect reaction time since attention is often biased towards objects nearer to observers. The depth effect has been so far ignored in this paper, although the search items were assigned heterogeneous depth values (see Materials and Methods) in Experiments 2–3, as well as in Experiment 1’s session shown in Figure 3B. Input luminance contrasts can also affect reaction times, since a stronger luminance contrast makes an input item more attractive to attention.

Experiment 4 was designed to examine the effects of these multiple stimulus factors mentioned above. It was a modified version of Experiment 3, using the same equipment setup, had the same task to search for a letter ‘T’ among letter ‘L’s. Each session randomly interleaved one baseline condition, in which all search items had zero ocularity O=0, and nine DC conditions in which only the target ‘T’ had non-zero ocularity *O*. These nine DC conditions differed from each other in terms of the ocularity magnitude |O| of the target and in whether this non-zero |O| was static or whether this |O| was transiently present for the initial period ΔT=0.1 or 0.5 s. When the target’s non-zero |O| was transient, it decayed smoothly to zero over the duration between ΔT to ΔT+0.1 s from the search array onset. The smooth decay rather than an abrupt disappearance was designed to eliminate the perception of the input changes. None of the nine observers for Experiment 4, including the sole non-naive observer (the author), noticed any changes (e.g., blinking or movements) associated with any search item during the visual search.

In addition, each letter in the search array was independently assigned a random value in its binocular input summation $C_{L}+C_{R}$, making the letter array appear heterogeneous in luminance contrast. In each trial, the $C_{L}+C_{R}$ for the target ‘T’ was neither the maximum nor minimum of all the $C_{L}+C_{R}$’s. Averaged across the trials, the $C_{L}+C_{R}$ for the target was the same as the average $C_{L}+C_{R}$ for the non-target ‘L’s. This design aimed to reduce any conspicuousness that might arise from any lustre percept of the ocularity singleton. Among the eight naive observers, only one noticed that in occasional trials, the target letter appeared to have a layer of glaze on it. After the experimental data taking, some observers were invited to view the search stimuli from the static DC conditions leisurely and were asked if they perceived lustre on the target letter ‘T’. Typically, lustre was not perceived when the target’s |O|=1/5, but was perceived when this |O|=3/5 [14].

Experiment 4 also differed from Experiment 3 by having lighter letters on a darker grey background, instead of dark letters on a lighter background. This was so that an ocularly unbalanced item should appear to have approximately the same perceived contrast as an ocularly balanced item with the same binocular summation $C_{L}+C_{R}$ of input contrasts [19].

It is worth noting that, in each session of this experiment, the ocularly unbalanced singleton was encountered in 90% of the trials and was always the target whenever it was present. In contrast, in each session of Experiments 1–3, at least one third of the trials had no ocularity singleton; the target was the ocularity singleton in no more than 50% of the trials; and in some trials, the ocularity singleton was not the target. Hence, Experiment 4 may be more likely to make observers associate, consciously or unconsciously, an ocularity contrast with the target. In addition, in this experiment, letting attention be drawn towards the ocularity singleton (rather than resisting the attentional attraction) should improve the task performance.

Note that the ocularity *O* for all the non-targets was zero in Experiment 4. In contrast, this *O* was non-zero for all the non-targets in Experiments 2–3 and was the negative of the *O* for the ocularity singleton. Therefore, in the DC conditions, given a target’s |O|, the ocularity contrast between the target and the background items in Experiment 4 was only half of that in Experiments 2 and 3.

**Figure 10:**
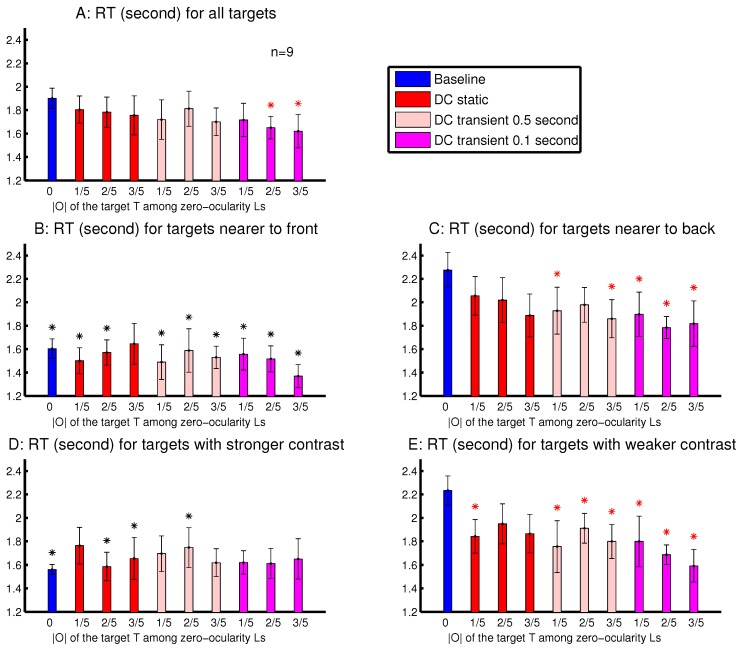
Effects arising from the target’s depth, luminance contrast, ocularity contrast, and the duration of the ocularity contrast in the task to find a target letter ‘T’ among ocularly balanced letter ‘L’s. Manual RT results are averaged across n=9 observers in sessions interleaving ten conditions, each defined by the ocularity magnitude |O| of the target ‘T’ and by whether and how transient this |O| was (when it was non-zero). Each input letter had a random depth (with a random disparity value within $\left(-0.21^{\circ },0.21^{\circ }\right)$, except for the zero-disparity items in the left most and right most columns of the search array) and a random (binocular summation of) luminance contrast $C_{L}+C_{R}$. (**A**) RT for each target’s |O| condition, averaged across observers regardless of the target’s depth and luminance contrast. (**B**,**C**) as in (A), except that each is only for trials with the near-targets (B) or the far-targets (C). (**D**,**E**) as in (A), except that each is only for trials with the targets having a stronger (D) or weaker (E) luminance contrast $C_{L}+C_{R}$. In each plot, a red ‘*’ above a data point from a DC condition (with target’s |O|>0) indicates that this RT was significantly different from the RT of the baseline condition (with target’s O=0) in the same plot. In (B) or (D), respectively, a black ‘*’ above each RT data point indicates a significant depth effect (B) or luminance contrast effect (D), when this RT was significantly different from the corresponding RT (in the condition having the same magnitude and temporal characters for the target’s |O|) in (C) or (E). All observers had normal depth vision, and one was not naive to the purpose of the study.

The observations from Experiment 4 are shown in Figure 10. The $RT_{\mathrm{DC}}$ was significantly shorter than $RT_{\mathrm{baseline}}$ when the target’s |O| was sufficiently strong and transient (Figure 10A). This is consistent with the idea, suggested by observations in Figure 8 and Figure 9, that an ocularly unbalanced target among ocularly balanced non-targets may repel observers unless the target’s non-zero ocularity is transient. However, unlike in sessions depicted in Figure 8 and Figure 9, observers in Experiment 4 did not perceive the transient changes by the disappearance of the ocularity contrast. Therefore the attentional cueing effect in Experiment 4 should be due to saliency by the ocularity singletons and not to any perception of input changes.

To analyse the depth effect, each target was categorized as a far-target or a near-target, when its distance from the viewer (by its binocular disparity) was farther or nearer than 50% of the letters in the search array. The attentional cueing effect by the ocularity contrast was much more evident for the far-targets than the near-targets (Figure 10B,C). In general, the far-targets evoked substantially longer RTs than the near-targets, particularly in the baseline condition. Hence, in the DC conditions, the natural attentional bias against the far-targets can be partially overcome by the saliency from the ocularity contrast. When heterogeneous depth values were also randomly assigned to search items in Experiments 2–3 and in Experiment 1’s session shown in Figure 3B, depth effects were also present, but were weaker. This is likely because these previous sessions had binocularly unbalanced inputs for all the search items, targets and non-targets. It is known that depth acuity is better when visual inputs for objects are binocularly balanced [20].

To analyse the effects from the luminance contrast, each target was categorized as to have a strong or a weak contrast, when its $C_{L}+C_{R}$ was larger or smaller than 50% of all the $C_{L}+C_{R}$’s (for all the letters) in a trial. Figure 10D,E shows that the attentional cueing effect by the ocularity contrast was much more evident for targets with weaker luminance contrasts $C_{L}+C_{R}$. These targets required longer RTs, particularly in the baseline condition. Hence, saliency by ocularity contrast in the DC conditions also helped to partially overcome the attentional bias against these weaker-contrast inputs. Together, Figure 10B–E indicates that ocularity contrast can help particularly visual inputs that are farther away from the viewers or are weaker in luminance contrast to overcome their weakness in attracting attention.

## 4. Discussion

This paper reports the following findings. First, saliency in an eye-of-origin singleton [8,9] can be generalized to saliency by an input singleton that is unique in the ocularity feature, which is defined as the relative difference between the left-eye input and the right-eye input for a visual input item. This saliency enables the singleton to attract attention exogenously, thus shortening the RTs to a search target when the singleton is also the target or prolonging the RTs by distracting attention away from the target towards the singleton far from the target. Second, an ocularity singleton can attract attention even when its ocularity distinction is very brief, consistent with the fact that exogenous attraction to attention acts quickly [17,18,21]. Third, saliency of an ocularity singleton is analogous to saliency of a colour singleton in the following sense. Just as the saliency of a colour singleton increases with the colour contrast between this singleton and background items, the saliency of an ocularity singleton also increases with the ocularity contrast between this singleton and the background items. Fourth, saliency of an ocularity singleton differs from saliency of a colour singleton in the following sense. When the visual inputs for an ocularity singleton are ocularly unbalanced, while the background inputs are ocularly balanced, this singleton can appear perceptually lustrous. Such a percept evokes avoidance behaviour in observers and inhibits the shifts of gaze towards the singleton. This inhibition masks the saliency effect behaviourally. In the colour analogue, an imbalance between inputs from different cones mainly makes the input appear colourful, but not discouraging, whether or not the background items are colourful or colourless. Fifth, the masked saliency of an ocularity singleton due to its evoked lustre percept could be somewhat unmasked by making the ocularity contrast very brief. This is done by making the imbalance between the input strengths to the two eyes very brief. The briefness is assumed to prevent the completion of the perceptual process towards the percept (of lustre) while allowing the fast-acting saliency process. Sixth, attentional attraction by ocularity contrast can help to make objects at farther distances from viewers or objects with weaker luminance contrasts partially overcome their disadvantage in attracting attention.

### 4.1. Neural Substrates of Saliency for Exogenous Attentional Attraction

The above findings provide additional evidence for the hypothesis [22,23] that the primary visual cortex (V1) creates a bottom-up saliency map to guide attention exogenously; see a review [1]. According to this hypothesis, the activity of the most activated V1 neuron responding to a given visual location represents the saliency of that location, and therefore, the receptive field location of the most activated V1 neuron by a visual scene is the most salient location in that scene. The neural mechanism for generating saliency signals is iso-feature suppression [22], so that nearby V1 neurons tuned to similar visual features (e.g., neurons preferring similar orientations or colours) suppress each other’s activities. Consequently, when a visual scene contains (e.g.,) a uniquely vertical bar in a background of horizontal bars, the strongest response to the vertical bar is higher than that to the horizontal bars since the neurons tuned to and responding to the vertical bar escape the iso-orientation suppression experienced by neurons tuned to and responding to the horizontal bars. This makes the location of the vertical bar most salient in this scene. In addition to iso-orientation suppression, other examples of iso-feature suppression include iso-colour suppression [24] and iso-motion-direction [25] suppression. Hence, analogously, the location of a uniquely red dot among green dots is salient, so is the location of a left-moving item in a background of right-moving ones. This hypothesis accounts for many phenomena of saliency behaviour, particularly those in visual search and texture segmentation [2,4,5,22,23,26,27,28]. Furthermore, it provided some testable predictions, including a quantitative prediction without any free parameters [29], that have been experimentally tested and confirmed subsequently. These experimental tests used visual psychophysics [29,30,31,32,33] or functional magnetic resonance imaging (fMRI) and event related potentials (ERPs) measured by electroencephalography (EEG) [34].

One particular theoretical prediction directly relevant to the current study is that the location of an eye-of-origin singleton should also be salient, even though the eye-of-origin feature is barely visible to perception. This prediction arises since iso-feature suppression in V1 also applies to the eye-of-origin feature [35]; hence, an eye-of-origin singleton in a background of items arising from the other eye-of-origin should also evoke a relatively less suppressed V1 response. The experimental confirmation [8,9] of this highly surprising prediction provided a hallmark of the saliency map in V1 since the eye-of-origin information was largely confined to V1 [36,37,38] among neocortical areas. The current study adds to the previous work by extending the eye-of-origin feature from being a discrete and binary value about the eye of origin to being an analog value using the ocularity feature defined in this paper.

### 4.2. Ocularity and Depth in Natural Inputs

Since the ocularity feature applies to general inputs, whether it is monocular, binocular, or ocularly unbalanced, it is more suitable than the binary eye-of-origin value to apply to natural inputs. Hence, a natural visual object has not only a depth feature value, but also an ocularity feature value. This is so at least when ocularity is measured on a coarse scale. In any case, a coarse scale is more suitable for the functional role of saliency, which is more important for the spatially low-resolution peripheral visual field, to which the gaze shifts guided by a saliency map of the visual field. Since inputs from the two eyes are highly correlated [39], natural inputs tend to have near-zero ocularity values. Hence, visual locations with significant ocularity contrasts tend to be those with substantially non-zero ocularity values, such as where input depth deviates from the plane of fixation. More generally, locations of significant ocularity contrasts are likely to be where object depth deviates from the average depth values of the contextual surround. Therefore, these locations are likely salient.

The findings in the current study (Figure 10) suggest that attention guided by saliency due to ocularity contrast can help overcome the attentional bias against farther objects or objects having a weaker luminance contrast. Saliency is computed in V1 and is fast acting [17,18,21], while depth seems to be computed beyond V1 along the visual pathway [40,41,42,43,44,45] and takes time to develop [46]. Binocular correspondence necessary for computing object depths does not seem to be established in V1 [40]. It would not be surprising that observers shift attention guided by saliency arising from ocularity contrasts before they perceive the depths of objects at the destination of the attentional shifts. This is not so surprising since attention or gaze can shift towards an eye-of-origin singleton without perceiving the eye-of-origin feature [8,9]; gaze also shifts to objects before objects are recognized [47,48,49].

### 4.3. Ocularity Feature and V1 Representation

The ocularity feature $s_{L}-s_{R}$ measures the relative difference between visual inputs to the two eyes. For efficient coding of visual inputs, V1 neurons represent binocular inputs in two decorrelated channels: binocular summation and binocular difference [39]. Signals in these two channels are multiplexed in V1’s population neural responses. Some monocular cells in V1 may be seen as signalling the binocular difference: although visual inputs to the “silent” eye alone do not evoke neural responses (beyond the near-zero spontaneous activities), they often suppress the neural responses when the other eye also receives visual inputs [50,51]. These ocular difference cells are useful for signalling non-zero depth [39,52]. These neurons should be useful to signal saliency from ocular contrasts.

V1 neurons that signal the binocular input difference, such as the monocular cells that are suppressed by inputs to their “silent” eye, can also be tuned to other visual features such as spatial orientation. Hence, in addition to signalling saliency and evoking the percept of lustre [12,13], the ocular difference channel in V1 can also convey non-ocularity information such as orientation, motion direction, colour texture [53], and even face identity or gender [54,55,56].

### 4.4. Interactions between Visual Perception and Visual Saliency

Saliency effects are much more evident for a zero-ocularity singleton among non-zero ocularity items than a non-zero ocularity singleton among zero-ocularity items; see Figure 7. Wolfe and colleagues [7,57] found an asymmetry in the opposite direction: it is easier to find a lustrous item among non-lustrous ones than the reverse (in their experiments with a non-interleaved block design). Paffen and colleagues [58] found a stronger asymmetry that it is easier to search for an ocularly conflict item among ocularly non-conflict items than the reverse. In both of the previous studies, the ocularity feature, in terms of ocular balance or conflict, defined the target of the visual search. In comparison, the current study does not define the target by the ocularity feature (see also [57]). The opposite directions of asymmetry observed in different studies demonstrate the importance of top-down, task-dependent, factors in overall behaviour. Saliency is an exogenous factor and is restricted to visual selection (looking), but not visual perception (seeing and recognition). Meanwhile, behaviour is typically the net effect of saliency, perception, and other top-down factors, and one needs to dissect behaviour to identify the saliency effects. The complexity of the interaction between the visual processes for looking and seeing calls for many more future investigations [1,53].

## 5. Conclusions

Saliency by contrast in eye-of-origin is extedned to saliency by contrasts in ocularity; and saliency increases with the magnitude of these contrasts. Hence, a visual location with a strong ocularity contrast attracts attention regardless of its task relevance, in support of the idea that the primary visual cortex creates a saliency map to guide attention exogenously [22,23].

Behavioural manifestation of saliency by ocularity contrasts may be masked by percepts evoked by input ocularity characters. Since the processes for exogenous attentional attraction act more quickly than the processes for seeing or perception, making the ocularity contrast sufficiently brief can help reduce the masking.

## References

[1] Zhaoping L. (2014). Understanding Vision: Theory, Models, and Data.

[2] Treisman A.M., Gelade G. (1980). A feature-integration theory of attention. Cognit. Psychol..

[3] Desimone R., Duncan J. (1995). Neural mechanisms of selective visual attention. Annu. Rev. Neurosci..

[4] Wolfe J., Cave K., Franzel S.L. (1989). Guided search: An alternative to the feature integration model for visual search. J. Exp. Psychol. Hum. Percept. Perform..

[5] Duncan J., Humphreys G. (1989). Visual search and stimulus similarity. Psychol. Rev..

[6] Wolfe J.M., Pashler H. (1998). Visual search, a review. Attention.

[7] Wolfe J., Franzel S. (1988). Binocularity and visual search. Percept. Psychophys..

[8] Zhaoping L. (2008). Attention capture by eye of origin singletons even without awareness—A hallmark of a bottom-up saliency map in the primary visual cortex. J. Vis..

[9] Zhaoping L. (2012). Gaze capture by eye-of-origin singletons: Interdependence with awareness. J. Vis..

[10] Zhaoping L. (2010). Ocularity as a basic visual feature dimension for bottom-up attentional attraction. Perception.

[11] Zhaoping L. (2014). Ocular contrast speeds up an inefficient search for a T among L’s which appear in non-uniform depth. Perception.

[12] Helmholtz H.V. (1924). Treatise on Physiological Optics (J.P.C. Southhall, Trans. Original Work Published 1909).

[13] Anstis S.M. (2000). Monocular lustre from flicker. Vis. Res..

[14] Formankiewicz M.A., Mollon J. (2009). The psychophysics of detecting binocular discrepancies of luminance. Vis. Res..

[15] Malkoc G., Kingdom F.A. (2012). Dichoptic difference thresholds for chromatic stimuli. Vis. Res..

[16] Georgeson M.A., Wallis S.A., Meese T.S., Baker D.H. (2016). Contrast and lustre: A model that accounts for eleven different forms of contrast discrimination in binocular vision. Vis. Res..

[17] Nakayama K., Mackeben M. (1989). Sustained and transient components of focal visual attention. Vis. Res..

[18] Müller H.J., Rabbitt P.M. (1989). Reflexive and voluntary orienting of visual attention: Time course of activation and resistance to interruption. J. Exp. Psychol. Hum. Percept. Perform..

[19] Baker D.H., Wallis S.A., Georgeson M.A., Meese T.S. (2012). Nonlinearities in the binocular combination of luminance and contrast. Vis. Res..

[20] Schor C., Heckmann T. (1989). Interocular differences in contrast and spatial frequency: effects on stereopsis and fusion. Vis. Res..

[21] Van Zoest W., Donk M. (2006). Saccadic target selection as a function of time. Spat. Vis..

[22] Li Z. (1999). Contextual influences in V1 as a basis for pop out and asymmetry in visual search. Proc. Natl. Acad. Sci. USA.

[23] Li Z. (2002). A saliency map in primary visual cortex. Trends Cognit. Sci..

[24] Wachtler T., Sejnowski T., Albright T. (2003). Representation of colour stimuli in awake macaque primary visual cortex. Neuron.

[25] Jones H., Grieve K., Wang W., Sillito A. (2001). Surround suppression in primate V1. J. Neurophysiol..

[26] Li Z. (1999). Visual segmentation by contextual influences via intra-cortical interactions in primary visual cortex. Netw. Comput. Neural Syst..

[27] Li Z. (2000). Pre-attentive segmentation in the primary visual cortex. Spat. Vis..

[28] Zhaoping L. (2003). V1 mechanisms and some figure-ground and border effects. J. Physiol. Paris.

[29] Zhaoping L., Zhe L. (2015). Primary visual cortex as a saliency map: A parameter-free prediction and its test by behavioural data. PLoS Comput. Biol..

[30] Zhaoping L., Snowden R. (2006). A theory of a saliency map in primary visual cortex (V1) tested by psychophysics of colour-orientation interference in texture segmentation. Vis. Cognit..

[31] Zhaoping L., May K. (2007). Psychophysical tests of the hypothesis of a bottom-up saliency map in primary visual cortex. PLoS Comput. Biol..

[32] Koene A., Zhaoping L. (2007). Feature-specific interactions in salience from combined feature contrasts: Evidence for a bottom-up saliency map in V1. J. Vis..

[33] Jingling L., Zhaoping L. (2008). Change detection is easier at texture border bars when they are parallel to the border: Evidence for V1 mechanisms of bottom-up salience. Perception.

[34] Zhang X., Zhaoping L., Zhou T., Fang F. (2011). Neural activities in V1 create a bottom-up saliency map. Neuron.

[35] DeAngelis G.C., Freeman R.D., Ohzawa I. (1994). Length and width tuning of neurons in the cat’s primary visual cortex. J. Neurophysiol..

[36] Hubel D., Wiesel T. (1968). Receptive fields and functional architecture of monkey striate cortex. J. Physiol..

[37] Burkhalter A., Van Essen D.C. (1986). Processing of colour, form and disparity information in visual areas VP and V2 of ventral extrastriate cortex in the macaque monkey. J. Neurosci..

[38] Zeki S.M. (1978). Uniformity and diversity of structure and function in rhesus monkey prestriate visual cortex. J. Physiol..

[39] Li Z., Atick J.J. (1994). Efficient stereo coding in the multiscale representation. Netw. Comput. Neural Syst..

[40] Cumming B.G., Parker A.J. (2000). Local disparity not perceived depth is signalled by binocular neurons in cortical area V1 of the macaque. J. Neurosci..

[41] Bakin J., Nakayama K., Gilbert C.D. (2000). Visual responses in monkey areas V1 and V2 to three-dimensional surface configurations. J. Neurosci..

[42] Von der Heydt R., Peterhans E., Baumgartner G. (1984). Illusory contours and cortical neuron responses. Science.

[43] Von der Heydt R., Zhou H., Friedman H.S. (2000). Representation of stereoscopic edges in monkey visual cortex. Vis. Res..

[44] Qiu F., von der Heydt R. (2005). Figure and ground in the visual cortex: V2 combines stereoscopic cues with gestalt rules. Neuron.

[45] Janssen P., Vogels R., Liu Y., Orban G. (2003). At least at the level of inferior temporal cortex, the stereo correspondence problem is solved. Neuron.

[46] Zhaoping L., Guyader N., Lewis A. (2009). Relative contributions of 2D and 3D cues in a texture segmentation task, implications for the roles of striate and extrastriate cortex in attentional selection. J. Vis..

[47] Zhaoping L., Guyader N. (2007). Interference with bottom-up feature detection by higher-level object recognition. Curr. Biol..

[48] Oliveri M., Zhaoping L., Mangano G., Turriziani P., Smirni D., Cipolotti L. (2010). Facilitation of bottom-up feature detection following rTMS-interference of the right parietal cortex. Neuropsychologia.

[49] Zhaoping L., Frith U. (2011). A clash of bottom-up and top-down processes in visual search: the reversed letter effect revisited. J. Exp. Psychol. Hum. Percept. Perform..

[50] Poggio G., Fischer B. (1977). Binocular interaction and depth sensitivity in striate and prestriate cortex of behaving rhesus monkey. J. Neurophysiol..

[51] Livingstone M., Hubel D. (1984). Anatomy and physiology of a colour system in the primate visual cortex. J. Neurosci..

[52] Kingdom F., May K., Hibbard P. Stereoscopic depth pereption is differentially affected by adaptation to binocularly correlated versus binocularly anti-correlated noise. Presented at the 40th European Conference on Visual Perception.

[53] Zhaoping L. (2017). Feedback from higher to lower visual areas for visual recognition may be weaker in the periphery: Glimpses from the perception of brief dichoptic stimuli. Vis. Res..

[54] May K., Zhaoping L., Hibbard P. (2012). Perceived direction of motion determined by adaptation to static binocular images. Curr. Biol..

[55] May K.A., Zhaoping L. (2016). Efficient coding theory predicts a tilt aftereffect from viewing untilted patterns. Curr. Biol..

[56] May K., Zhaoping L. (2016). Face gender adaptation from random noise adaptors: A surprising prediction of Li and Atick’s efficient binocular coding theory. J. Vis..

[57] Zou L., Utochkin I.S., Liu Y., Wolfe J.M. (2017). Binocularity and visual search—Revisited. Atten. Percept. Psychophys..

[58] Paffen C.L., Hooge I.T., Benjamins J.S., Hogendoorn H. (2011). A search asymmetry for interocular conflict. Atten. Percept. Psychophys..

